# Peer support workers as a bridge: a qualitative study exploring the role of peer support workers in the care of people who use drugs during and after hospitalization

**DOI:** 10.1186/s12954-021-00467-7

**Published:** 2021-02-16

**Authors:** Robin Lennox, Larkin Lamarche, Tim O’Shea

**Affiliations:** 1 Department of Family Medicine, McMaster University, Hamilton, Canada; 2 Division of Infectious Diseases, Department of Medicine, McMaster University, Hamilton, Canada

**Keywords:** Harm reduction, Peer support, Treatment models/programs

## Abstract

**Background:**

To describe the key qualities and unique roles of peer support workers in the care of people who inject drugs during and after hospitalization.

**Methods:**

We conducted a qualitative study. Key stakeholders were recruited including: people who use drugs who had been hospitalized, healthcare team members, peer support workers, and employers of peer support workers. Data were collected from 2019 to 2020 using semi-structured interviews that were audio-recorded, transcribed, and analyzed thematically.

**Results:**

Fourteen participants were interviewed: 6 people who use drugs who had been hospitalized, 5 healthcare team members, 2 peer support workers, and 1 employer of peer support workers. At the core of the data was the notion of peer workers acting as a bridge. We found four themes that related to functions of this bridge: overcoming system barriers, advocacy, navigating transitions within the healthcare system, and restoring trust between HCPs and PWUD. We found two themes for building a strong bridge and making the role of a peer support worker function effectively (training and mentorship, and establishing boundaries). We found three themes involving characteristics of an effective peer worker (intrinsic qualities, contributions of shared experiences, and personal stability).

**Conclusion:**

Peer support workers are highly valued by both people who use drugs and members of the healthcare team. Peer support workers act as a bridge between patients and healthcare providers and are critical in establishing trust, easing transitions in care, and providing unique supports to people who use drugs during and after hospitalization.

## Background

People who use drugs (PWUD) present to the emergency department and are hospitalized more often than the general population [1–3]. Hospitals have been identified as a “risk environment” for PWUD, who experience significant barriers to accessing health services [4, 5]. The stigma experienced by PWUD in hospital can result in delayed presentation to care, lack of trust in healthcare providers, higher rates of patient-initiated discharge, and hesitance to access acute care when needed [4–7].

Healthcare providers (HCPs) have also expressed feeling ill-prepared and reluctant to provide care for PWUD [8, 9]. This reluctance can manifest as avoidance and lack of engagement, often interpreted by PWUD as neglect and mistreatment [4, 5, 9, 10]. Many studies have shown that HCPs hold negative attitudes towards PWUD which can reinforce the stigma experienced by PWUD when accessing healthcare [9]. Thus, despite frequent interactions between PWUD and HCPs in acute care settings, significant barriers exist that impair the formation of a strong therapeutic alliance and adversely impact the hospital experiences of PWUD [10].

Integrating people with lived experience of drug use as partners in clinical care delivery in hospitals has been proposed as a potential de-stigmatizing intervention [7]. Peer support workers are individuals with lived experience who may take on formal or informal roles in the care of the clients they serve. With the advantage of shared experiences, peer support workers can more effectively engage hard-to-reach populations, including those complex mental health concerns [11]. The use of peer workers has been well studied in community-based models of care supporting individuals with severe mental illness [11, 12]. In one randomized-controlled trial in New Haven, Connecticut, the use of peer mentors for patients with severe mental illness was demonstrated to reduce hospital readmissions and total days in hospital over a period of nine months following the intervention [13]. The use of community-based peer workers for PWUD has also been shown to have favorable outcomes specific to substance use: reduced substance use, better retention in treatment, and improved relationships with treatment providers [14–17].

The role of peer workers for PWUD in hospital environments is an area of growing interest. The use of peer workers in the emergency department, particularly for people with opioid use disorder and those presenting with nonfatal opioid overdose, has been studied within various models of care [18] Within these settings, the core function of peer workers is often to assist patients in connecting to medication-assisted treatment and other community resources upon discharge from the ED [18]. However, literature on the role of peer support workers in supporting PWUD while hospitalized is sparse. To our knowledge, two studies have been published that specifically examine the role of peer workers for PWUD while admitted to hospital [8, 14]. Both studies arose from the integration of “peer mentors” into an inpatient addiction consultation team (IMPACT) in Portland, Oregon [8, 14]. One study highlighted strategies to successfully integrate peer mentors into the healthcare team and identified that clear role definition, structured hiring and retention process, and ongoing training were critical [14]. The second study evaluated the role of peer mentors and found that they were considered valuable additions to the healthcare team, assisted in engaging patients, and acted as cultural brokers between patients and HCPs [8].

Despite the well-established challenges faced by PWUD when hospitalized and the limitations of traditional HCPs to provide care to this patient population, there remains a paucity of literature on the use of peer support workers with lived experience of drug use to support PWUD in acute care settings. This study aimed to address this gap by exploring: (1) the qualities that make a good peer support worker for PWUD during and after hospitalization for acute illness; (2) the essential functions of a peer support worker for hospitalized PWUD; and (3) facilitators and barriers for integrating peer support workers into the hospital setting.

## Methods

### Study setting and design

The study took place between March and September 2019 in an urban center in Southwestern Ontario, Canada. Patient participants were recruited from two academic hospitals in Hamilton, Ontario. This was a qualitative study.

### Participants

Fourteen participants were interviewed: 6 hospitalized PWUD, 5 members of the healthcare team (two family physicians, general internist, infectious diseases specialist, cardiologist), two peer support workers (with previous training and experience working in community-based health outreach and harm reduction agencies), and one employer of peer support workers.

## Research team

The research team was composed of three individuals. The first author is a family physician with expertise in addiction medicine. She coded 100% of the transcripts. The second author was a research associate with over 10 years of research experience and 5 years of healthcare research. They also have experience with qualitative research designs and interviewing participants. They conducted and coded 100% of the interviews. The third author is an infectious disease specialist with expertise in addiction medicine and low-barrier care for PWUD. He helped with the contextualization of the results and challenged the thematic map. All authors believe in a harm reduction and person-centered approach to providing care to PWUD.

### Interview guide

The first and second authors developed the interview guide. Questions were grounded in the study’s research questions and practically driven; that is, they were framed such that information could be used in the planning and development phases of a peer-worker intervention. Interviews guides had some questions that were tailored to each participant group. For example, a question about hiring practices was asked to peer support worker employers but not to patients. The first interview (that was with a patient) was treated as a pilot interview. It was deemed successful based on the depth of information gathered, and no changes to the interview guide were made. We also asked each participant at the end of their interview; if there were questions, we should be asking; all participants did not suggest any additional questions.

### Procedure

Upon institutional research ethics board approval, participants were recruited using purposive sampling procedures to gather in-depth information specific to our research questions [19]. We recruited patients, HCPs, peer support worker employers, and peer support workers. Patients were introduced to the study and invited to be contacted by the research team during their hospital admission through the inpatient addiction medicine team. One-on-one interviews for patients were conducted face to face. HCPs, peer support worker employers, and peer support workers were informed about the study through the inpatient addiction medicine team at the hospital or the research team’s network connections within the community and connected to the interviewer. One-on-one interviews for HCPs, peer support worker employers, and peer support workers were conducted by phone or face to face. Duration of the interviews ranged from 15 to 30 min and was audio-recorded. Participants were provided with a $20 gift card. After the 14th interview, it was decided that the richness of the meaning was sufficient to answer our research questions and recruitment was ended [20].

### Data analysis

A professional transcription company was used to transcribe audio recordings verbatim. We used (reflexive) thematic analysis [21] to identify, analyze, and report patterns or themes in the interview data. We inductively coded the data and followed the six phases of thematic analysis outlined by Braun and Clarke [21]. Specifically, we familiarized ourselves with the data by reading and re-reading the transcripts. Next, we generated initial codes which were inductive and grounded in the data. We searched for themes by grouping codes together and then reviewed potential themes (e.g., finalizing the thematic map). In the fifth phase, we defined and named themes. Finally, we produced the final report. In phases five and six, we refined the thematic map in meetings between the first and second authors. These meetings involved challenging each other on the reconstruction of the data whereby we would visit the data to confirm support for our themes. Coding and analysis were done not with the intention of looking for differences between participant groups. Analysis was primarily grounded in a pragmatic framework [22]. Within a pragmatic framework, the approach to the research design and rigor was driven by the research question; thus, the importance is placed on the continuity between the research question, research design, and analysis.

We followed Lincoln and Guba’s [23] and Tracy’s [24] recommendations for ensuring the trustworthiness, authenticity, and credibility of the data. For credibility, we used purposive sampling—participants who could speak to the topic intimately, providing rich information to answer our research questions. For authenticity, direct quotations were used in the results. Credibility was fostered by having two people code and analyze the data independently. The first and second authors met to discuss interpretations and initial theme development after the first five interviews, and again after all the interviews. In each meeting, the coders discussed discrepancies until consensus was reached. These meetings also involved refinement of the thematic map.

## Results

All participants had interacted with peer support workers to different extents and/or were aware of the peer support worker role, but most had some past experience with peer workers that informed their responses. All six PWUD interviewed said that they would welcome involvement of peer workers in their care.

### Thematic map overview

**Fig. 1:**
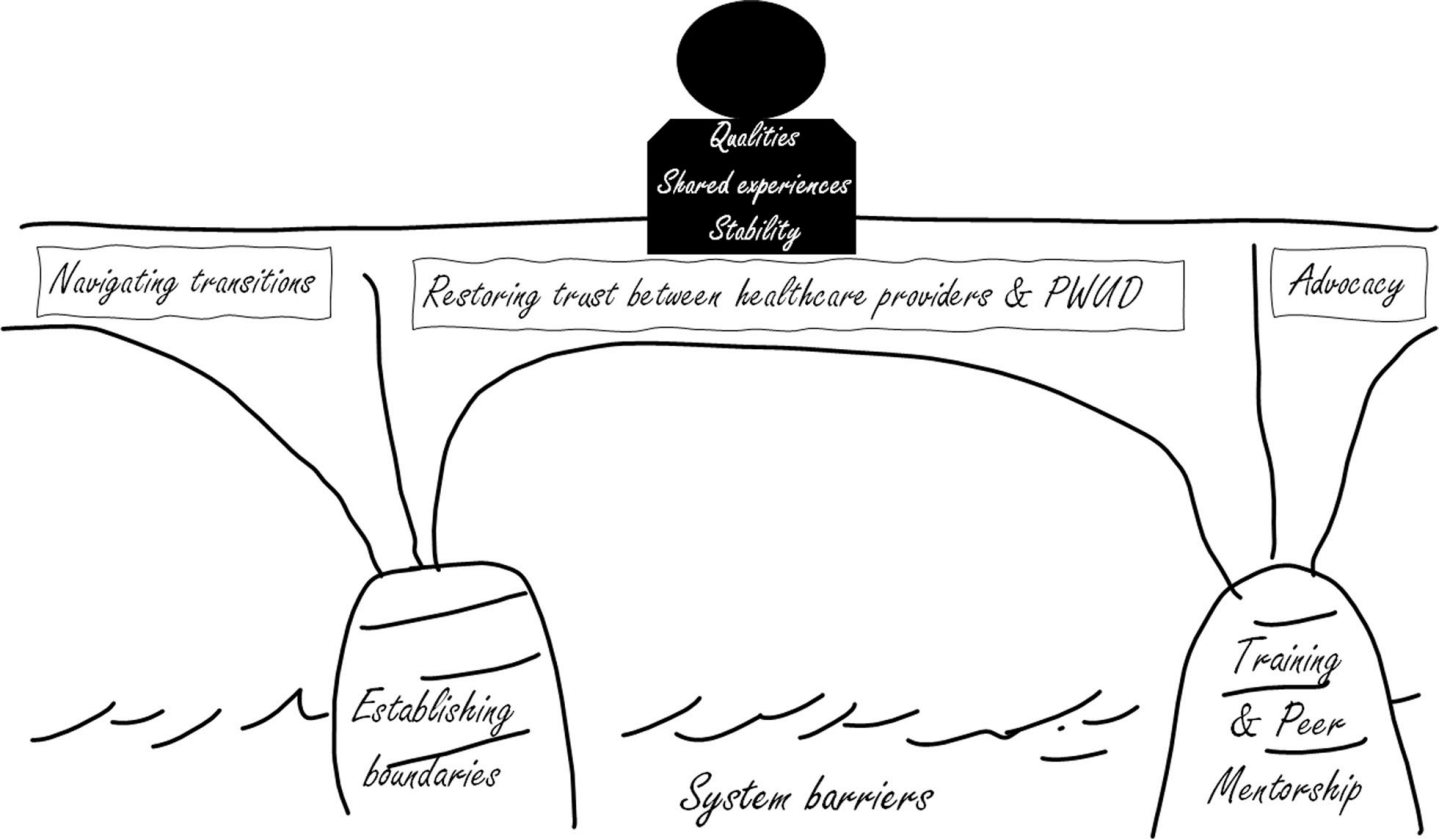
Thematic map

As demonstrated by the thematic map (Fig. 1), at the core of the data was the notion of the peer worker acting as a bridge. We found four themes that related to functions of this bridge: overcoming system barriers, advocacy, navigating transitions within the healthcare system, and restoring trust between HCPs and PWUD. We found two themes for building a strong bridge and making the role of a peer worker function effectively (training and mentorship and establishing boundaries). We also found three themes involving characteristics of an effective peer worker (intrinsic qualities, contributions of shared experience, and personal stability).

#### The Core: Peer support workers as a bridge

Central to the data was the concept of peer support workers as a “bridge” and served as an anchor in our analysis. The word “bridge” was mentioned in various ways by HCPs, peer support workers, and employers of peer support workers within the study to illustrate their importance. For example, “bridge” was used to describe how a peer worker could facilitate a discharge plan (P9, HCP), or serve as a connection between the patient and the healthcare team, or with community resources (P10, P11, P12, HCPs), or to bridge gaps in communication and information (P11, HCP).

### Functions of the bridge

Closely tied to the core concept of peer workers as a bridge were themes relating to the functions of peer workers for PWUD in hospital and after discharge: overcoming system barriers, advocacy, navigating transitions within the healthcare system, and restoring trust between HCPS and PWUD.

Participants used this analogy explicitly; “I [the peer worker] was also able to connect with people and be that bridge that bridged the gap between the academics, the medical people and the people who are using… That’s kind of the job of a peer support worker. We want to bridge the gap between these two because people have a lot of fear of hospitals and the medical industry, the medical scene” (P13, peer worker).

#### Overcoming systemic barriers

As a “bridge”, peer workers were felt to have the potential to help overcome systemic barriers faced by PWUD in hospitals, such as system trauma, stigma, and inflexibility. Participants paired the acknowledgement of these system barriers that PWUD have experienced with the notion that peer workers have a unique lens to offer, as they may have experienced similar barriers themselves. For example, P6 noted that “they [peer support workers] see that there's a value in connecting with the system, but they also recognize that it isn't easy, and that it can be very stigmatizing…So, I think that's where the value comes in; it's that bridge and that buffer between a system that can actually do harm in some ways and also benefit” (peer support worker employer).

Participants described acute care as a highly structured and inflexible environment that can cause tension between PWUD and HCPs, and one in which peer workers could assist in overcoming system rigidity. One patient described a conflict that arose with her nurse because she was not allowed to leave the ward to go outside for a cigarette: “nobody will compromise with me and I am lashing out because of it.” (P4) She went on to reflect that if there was a peer support worker involved in her care, they may have been able to work together differently:

“To get on the same page. And, you know, it’s something that the nurses and like people like me can’t do, to be honest; and something that a peer support worker and I probably could do, and we could compromise like that. (P4, patient)”

Both PWUD and HCPs felt that peer support workers could use their unique lens to be creative about solutions to overcome inflexibility, and could work with both patients and HCPs to make these changes work:

“let’s figure out a different way. If one way is not working, figure something else out… you know, a different solution.” (P4, patient)”

“all of our physicians are willing to think outside the box, but sometimes…we need some help coming up with the different ideas (P12, HCP).”

#### Advocacy

Many PWUD felt disempowered within the hospital system and thought that a peer worker would be able to advocate for their needs both in hospital and after discharge. One participant recounted her experience presenting to hospital:

“Like I don’t know if that whole—the first hospital I went to up in [city] was just horrid; like it’s almost like they didn’t believe me that I was having pain. Like I was having a lot of pain in my leg, that was because my body was full of infection and because I had a heart infection…it’s like they almost didn’t believe me and thought I was just there wanting drugs or something.” (P7, patient)

She went on to express that she would have felt better if someone like a peer worker was with her at the time who could “speak up for [her]” (P7, patient) and help advocate for her needs. All other PWUD reaffirmed this sentiment and described peer support workers as someone who could “push for you” (P5, patient) or “be my voice” (P4, patient).

As one PWUD identified, peer support workers having credibility as part of the healthcare team would allow them to advocate for PWUD more effectively with other HCPs:

“my credibility and maybe a peer support worker's credibility are two different things. Like, they could also be a voice, right. Because a lot of times the nurse is not willing to listen…there could be somebody there who has got a bit more credibility and can, you know, relate to them as well.” (P4, patient)

#### Navigating transitions within the healthcare system

A salient theme was the role of peer workers in bridging transitions from hospital to outpatient care. All HCPs felt that the immediate post-discharge period was the highest risk for adverse patient outcomes, often due to patients facing structural barriers (lack of housing, lack of transport, limited social supports) in their home environments that interfered with their ongoing engagement in care. PWUD also felt that upon leaving hospital, they would have limited awareness of the resources available to them and that a peer worker would be helpful: “They know more about what's in the neighborhood or your community…. I really don't know anything when I'm leaving here.” (P5, patient) Similarly, HCPs felt that once the patient left the hospital, they had limited ability to maintain contact and ensure good continuity of care.

In transitioning from hospital-based to community-based care, participants envisioned peer support workers acting as a “touch point” (P10, HCP) and providing continuity between settings. This navigation could be bidirectional: “…both the patient getting back in the healthcare system, if needed, and the healthcare system getting in touch with the patient.” (P9, HCP).

If given flexibility in their role and the opportunity to connect with patients in both hospital and community settings, peer workers could position themselves as “being a main bridge between those two times [hospital and community-based care] (P11).”

#### Restoring trust

Participants identified that “there is a distrust between… the community that we're dealing with and the medical system” (P10, HCP) and that when integrated effectively, peer support workers could act “as the bridge between the two” (P10, HCP).

“all sorts of different kind of longstanding historical pieces that I think have really contributed to the culture of making it a really unpleasant experience when people who use substances come into hospital. There’s a significant lack of trust for a lot of those reasons as well.”

Participants expressed that the lack of trust could impede the provision of adequate care and that for PWUD to feel comfortable discussing their substance use with the healthcare team, trust is essential.

“to help your patient, to get them to open up […] so you can give them proper care they have to be able to trust to talk to you, and especially about something so vital in their life” (P4, patient).

Having peer workers representing the community of PWUD within the healthcare team was noted to put patients at ease and begin to restore a sense of safety within healthcare spaces.

“[peer support workers] did a lot in terms of building trust for people to get in the door. Because I think what we realized really early was that people actually don’t feel really reassured by a doctor or nurse being around, but they feel really reassured by someone from their community being there and telling [them] that it’s a safe place.” (P8, HCP).

Peer support workers also identified rebuilding trust between patients and HCPs as being a key component of their role.

“… to build trust between doctors and people that […] may have had extremely bad experiences” (P13, peer worker).

One physician felt that the peer worker could lend them credibility by being “…someone who is part of the community, who is fairly known within the community who can kind of vouch for me.” (P10, HCP).

### Building a strong bridge: making the role of peer support worker function effectively

#### Ongoing training and peer mentorship

Participants emphasized the importance of adequate training for peer support workers to allow them to function effectively. Having lived experience is essential and informative, but without training on how to channel that lived experience to serve the needs of clients, peer support workers felt poorly resourced to fulfill their role:

“I wasn’t saying the right things even through my lived experience… I was like, I don’t know what to do in this situation, I don’t know how to help someone else, I don’t know what to say, you know, and how to keep them safe, and what is crossing boundaries and what isn’t.” (P13, peer worker)

In particular, participants highlighted the need for specific training about mental health and suicide prevention, especially when serving clients with complex mental health needs. Harm reduction training and guidance on boundary setting were also noted to be essential for peers working with PWUD.

Participants also emphasized the need for ongoing support and mentorship of peer support workers over the course of their employment. This can be achieved through “continual coaching” (P6, peer support worker employer) or the use of a designated peer mentor (distinct from a peer support worker) to provide ongoing guidance. In essence, this peer mentor role serves as a preceptor role for the peer support workers and is someone who may have additional training, experience, or knowledge. This ongoing mentorship was noted to be helpful as challenges unique to the peer worker role arise, such as boundary concerns and ethical issues.

#### Establishing boundaries

Many participants emphasized the critical role of establishing boundaries in peer support work. These boundaries are rooted in clearly defined expectations of the role. When describing what went wrong with a past initiative involving peer support workers, P6 said “you do have to be very clear about what your expectations are from the beginning…I don’t think there were clear expectations as to what was okay and what wasn’t okay [regarding the past initiative], and so then when things started happening that weren’t okay, people were confused, right, because that hadn’t been communicated that that wasn’t okay.” These predetermined expectations help set boundaries, which served a dual purpose: allowing the peer support worker to function effectively in their professional role, and mitigating the risk of social distress and burnout.

Especially if working within a community that they have lived in or have strong relationships with, peer support workers can encounter situations where maintaining professional boundaries is challenging:

“We’re going to have to be careful… about overlap between maybe social circles or communities from where the person with lived experience or the peer support worker comes from and the individuals that they’re helping to support because it’s important that they come with some shared perspective, but you don’t want it to feel not safe to either of them because there’s too much overlap between their original communities.” (P12, HCP)

The overlap between professional and personal communities was a challenge that peer workers recounted from past experiences; “I had to figure out my boundaries… it's a unique situation for a peer support worker. You know, a lot of these people that are coming in… you know them.” (P13) Even if previous relationships did not exist, peer support workers articulated that the unique therapeutic relationship formed between peer support workers and their clients could lead to blurring of professional boundaries:

“There's a certain inherent sort of trust between people that gets built, and it becomes a type of a friendship; but you have to be really careful to keep people that you're working with professionally. It's difficult, but you need to be able to keep that professional.” (P14)

If professional boundaries were not upheld, or if peer support workers were not given guidance on how to set these boundaries, they can be at risk for burnout. As per one peer support worker; “… it ended up becoming a problem for me because I had no space for myself, and I was getting overtired. Because like my job would never kind of end.” (P13) Maintaining boundaries was noted to be an essential component of self-care and sustainability for peer support workers; “Boundaries ties us in with self-care… if I can’t take care of myself, I cannot do my job. I can’t help people.” (P13, peer worker).

### Characteristics of an effective peer support worker

#### Intrinsic qualities

Participants listed many intrinsic qualities that a good peer support worker should possess. Desirable qualities included being reliable, open-minded, respectful, adaptable, and empathetic. Participants voiced a need for the peer support worker to be highly sociable, be able to form strong social connections, and to read people well: “you have got to be able to mirror the person that you're talking to” (P14, peer worker). A consensus formed among participants that the peer support worker must be an exceptional communicator, and more importantly—an excellent listener: “someone who listens, that takes time to listen… who genuinely cares” (P7, patient).

In terms of characteristics to avoid, being judgmental and closed-minded were noted as highly undesirable qualities: “not being able to understand the other side of the coin (P2, patient).”

#### Contribution of shared experiences

Patients clearly articulated the unique benefits of having a peer with lived experience involved in their care. Having a shared understanding of the challenges of substance use made PWUD feel more comfortable opening up to peer workers, and made peer workers more relatable members of the healthcare team.

“…if they have been there and done it themselves, they understand more than anybody else… it's easier to talk to somebody who understands and has been through it themselves, than somebody who has just read it out of a textbook.” (P3, patient)

Healthcare providers also recognized the benefits of this shared understanding. One provider commented, “I think it would be powerful to have somebody that can really be there with them and understand it in a different way than the rest of our care team can” (P12, HCP).

An additional benefit of integrating peer support workers with the traditional healthcare team was their unique lens in identifying potential triggering aspects of acute care for PWUD.

“I think somebody with lived experience, a peer support worker, might be able to understand how bothersome it might be when people are poking your arm or whatever again for bloodwork or having, you know, constant IV access as potentially a trigger for you for ongoing injection drug use.” (P12, HCP)

When asked about the importance of the peer worker’s lived experience mirroring their own, most PWUD felt that it was unnecessary for the peer worker to have used the same substances or have a shared substance use pattern as they did. As long as the peer worker had lived experience, the specifics of their substance use were less important, “It doesn't matter because it's all the same; it's an addiction.” (P5, patient).

#### Personal stability

When asked about whether peer workers being active or in remission from their substance use should be a consideration for the role, there was no consensus among participants. Rather, many participants brought up the concept of “stability” as being most important for a successful peer worker. Stability was conceptualized as the sum of many factors including: an individual’s substance use pattern, mental health support, housing stability, and access to social supports.

“I think in this role, if you’re there as a support person for someone else, you just have to have at least enough stability, whatever that means for you, to be able to be reliable and show up [for] someone else.” (P8, HCP)

Many participants felt that the role of peer worker is diverse, and depending on the setting they were working in, the level of personal stability required may differ. As one peer support worker said: “I think that there’s a place for everybody in peer support work.” (P13, peer worker).

In highly structured settings such as acute care, one participant felt that unstable substance use could potentially impact the peer worker; “in terms of their ability to fulfill their role on a regular basis” (P10, HCP).

Many participants felt that as long as the peer worker was “strong and stable enough that they can support other people,” (P9, HCP) there were no specific requirements as to where that individual should be in their personal arc of substance use. Instead, the most important requirement was that they be able to perform the duties of their role reliably. In determining this stability, one participant felt that the best gauge of readiness for the role would be peer worker self-assessment: “they're the ones that should be able to tell us where they are in their journey” (P11, HCP).

## Discussion

Our study adds to the growing evidence supporting the integration of peer support workers for PWUD in hospital settings. Specifically, our findings highlight that integration of peer support workers at the hospital level may not only offer tangible supports to PWUD while in hospital and after discharge (i.e., address service gaps), but also help to dismantle existing system barriers.

The analogy of peer support workers serving a bridge is consistent with findings from the limited studies available on peer workers in hospitals [8, 14]. A critical function of the peer role is to rebuild trust between PWUD and HCPs [8, 14], which was affirmed in our study. Other critical functions of peers as a “bridge” included advocacy and navigating transitions in care. The concept of “peer-facilitated care planning” has been described in the literature as being more inclusive of patient-identified needs, such as housing and employment [12]. Our findings suggest that peer-facilitated care planning could be particularly impactful during transitions in care, particularly for discharge planning.

The intensity of the hospital environment and working with acutely ill clients has been identified as a potential challenge for peer workers who may not have much experience in acute care settings [14]. Our findings affirmed that without supportive measures in place, peer workers are at risk of social distress and burnout, particularly if boundary setting is not enforced. Potential strategies to support the successful integration of peer workers identified in our study were largely consistent with those in the limited literature available: ensure appropriate and ongoing training, select candidates with qualities that lend themselves to the peer role, and provide meaningful supervision [14]. On the latter point, the employer of peer support workers highlighted the potential role of “peer mentors” in a supervisory capacity—someone who has previous experience in the peer role and who could provide ongoing guidance to peer workers. This layered approach to integrating peers has not yet been explored in the hospital setting, but could be potentially helpful in assisting peer workers in navigating the challenges unique to their role.

A common query in selecting peer support workers is whether or not there should be a requirement for remission from substance use prior to engaging in peer support work. In the only other published work addressing this in hospital-based peer workers, Englander et al. suggested that peer workers should be stable in their recovery, or have two years of continuous remission from substance use [14]. In our study, there was no consensus suggesting that peer workers must be in remission from substance use. Rather, a consensus formed suggesting that peer workers should have enough personal stability to allow them to reliably fulfill their role requirements, with stability being influenced by many factors, including housing, mental health, and social supports.

## Limitations

Despite our study’s contributions, some limitations should be noted. First, patients were interviewed during their hospitalization; thus, impressions of discharge were not based on their actual experience being discharged from hospital. Second, we used purposive sampling to gather in-depth information; however, other stakeholders, such as nurses, administrative staff, policy makers, may offer different (or peripheral) perspectives. Finally, our study was within the context of the publicly funded Canadian healthcare system. The peer support worker role may operate differently in other health systems and funding models.

## Conclusion

Hospitalization can be a critical moment in a patient’s healthcare journey, and one that is particularly challenging for PWUD who face system barriers and distrust when accessing health services [4, 5]. Our findings suggest that integrating peer support workers into hospital teams is a potential intervention that could combat these barriers for PWUD [7] and help to rebuild trust in the healthcare system. Guidance on how to successfully integrate peers within hospital care teams is sparse [14]. Our findings highlight the need for programs to ensure adequate training and mentorship, boundary setting, and the use of self-care practices to protect peer workers against distress. Our findings add to the growing support for the use of peer support workers for PWUD in hospital as a novel concept. Further research is needed to explore the impact of peer support workers on outcomes for hospitalized PWUD, such as rates of treatment completion, engagement in care after discharge, and hospital utilization patterns.

## Data Availability

Data sharing not applicable to this article as no datasets were generated or analyzed during the current study.

## Abbreviations

PWID: People who inject drugs
PWUD: People who use drugs
HCPs: Healthcare providers

## References

[1] Kerr T, Wood E, Grafstein E, Ishida T, Shannon K, Lai C (2005). High rates of primary care and emergency department use among injection drug users in Vancouver. J Public Health.

[2] Palepu A, Tyndall MW, Leon H, Muller J, O’Shaughnessy MV, Schechter MT (2001). Hospital utilization and costs in a cohort of injection drug users. CMAJ.

[3] Kendall CE, Boucher LM, Mark AE, Martin A, Marshall Z, Boyd R (2017). A cohort study examining emergency department visits and hospital admissions among people who use drugs in Ottawa, Canada. Harm Reduct J.

[4] McNeil R, Small W, Wood E, Kerr T (2014). Hospitals as a 'risk environment': an ethno-epidemiological study of voluntary and involuntary discharge from hospital against medical advice among people who inject drugs. Soc Sci Med.

[5] Chan Carusone S, Guta A, Robinson S, Tan TH, Cooper C, O’Leary B (2019). “Maybe if I stop the drugs, then maybe they’d care?”—hospital care experiences of people who use drugs. Harm Reduct J.

[6] Ti L, Ti L (2015). Leaving the hospital against medical advice among people who use illicit drugs: a systematic review. Am J Public Health.

[7] Sharma M, Lamba W, Cauderella A, Guimond TH, Bayoumi A (2017). Harm reduction in hospitals. Harm Reduct J.

[8] Collins D, Alla J, Nicolaidis C, Gregg J, Gullickson DJ, Patten A (2019). "If it wasn't for him, I wouldn't have talked to them": qualitative study of addiction peer mentorship in the hospital. J Gen Intern Med.

[9] van Boekel LC, Brouwers EP, van Weeghel J, Garretsen HF (2013). Stigma among health professionals towards patients with substance use disorders and its consequences for healthcare delivery: systematic review. Drug Alcohol Depend.

[10] Merrill JO, Rhodes LA, Deyo RA, Marlatt GA, Bradley KA (2002). Mutual mistrust in the medical care of drug users: the keys to the "narc" cabinet. J Gen Intern Med.

[11] Wright-Berryman JL, McGuire AB, Salyers MP (2011). A review of consumer-provided services on assertive community treatment and intensive case management teams: implications for future research and practice. J Am Psychiatric Nurses Assoc.

[12] Davidson L, Bellamy C, Guy K, Miller R (2012). Peer support among persons with severe mental illnesses: a review of evidence and experience. World Psychiatry.

[13] Sledge WH, Lawless M, Sells D, Wieland M, O'Connell MJ, Davidson L (2011). Effectiveness of peer support in reducing readmissions of persons with multiple psychiatric hospitalizations. Psychiatr Serv.

[14] Englander H, Gregg J, Gullickson J, Cochran-Dumas O, Colasurdo C, Alla J (2019). Recommendations for integrating peer mentors in hospital-based addiction care. Subst Abus.

[15] Bassuk EL, Hanson J, Greene RN, Richard M, Laudet A (2016). Peer-delivered recovery support services for addictions in the United States: a systematic review. J Subst Abuse Treat.

[16] Reif S, Braude L, Lyman DR, Dougherty RH, Daniels AS, Ghose SS (2014). Peer recovery support for individuals with substance use disorders: assessing the evidence. Psychiatr Serv.

[17] Liamputtong P (2013). Qualitative research methods. Chapter 1, Methodological frameworks and sampling in qualitative research.

[18] McGuire AB, Powell KG, Treitler PC, Wagner KD, Smith KP, Cooperman N (2020). Emergency department-based peer support for opioid use disorder: Emergent functions and forms. J Subst Abuse Treat.

[19] Patton MQ (2002). Qualitative research and evaluation methods.

[20] Braun V, Clarke V (2019). To saturate or not to saturate? Questioning data saturation as a useful concept for thematic analysis and sample-size rationales. Qual Res Sport Exerc Health.

[21] Braun V, Clarke V (2006). Using thematic analysis in psychology. Qual Res Psychol.

[22] Morgan DL (2014). Pragmatism as a paradigm for social research. Qual Inquiry.

[23] Lincoln YS, Guba EG (1986). But is it rigorous? Trustworthiness and authenticity in naturalistic evaluation. New Dir Program Eval.

[24] Tracy SJ (2010). Qualitative quality: Eight “big-tent” criteria for excellent qualitative research. Qual Inquiry.

